# Effects of Ultra-High-Pressure Jet Processing on Casein Structure and Curdling Properties of Skimmed Bovine Milk

**DOI:** 10.3390/molecules28052396

**Published:** 2023-03-06

**Authors:** Fei Xu, Lu Xue, Yanfeng Ma, Tianjiao Niu, Pei Zhao, Zijian Wu, Yanfa Wang

**Affiliations:** 1 Tianjin Key Laboratory of Food Biotechnology, College of Biotechnology and Food Science, Tianjin University of Commerce, Tianjin 300134, China; 2 Mengniu Hi-Tech Dairy (Beijing) Co., Ltd., Beijing 101107, China

**Keywords:** ultra-high-pressure jet processing, casein, protein structure, curdling properties

## Abstract

Ultra-high-pressure jet processing (UHPJ) is a new non-thermal processing technique that can be employed for the homogenization and the sterilization of dairy products. However, the effects on dairy products are unknown when using UHPJ for homogenization and sterilization. Thus, this study aimed to investigate the effects of UHPJ on the sensory and curdling properties of skimmed milk and the casein structure in skimmed milk. Skimmed bovine milk was treated with UHPJ using different pressures (100, 150, 200, 250, 300 MPa) and casein was extracted by isoelectric precipitation. Subsequently, the average particle size, Zeta potential, contents of free sulfhydryl and disulfide bonds, secondary structure, and surface micromorphology were all used as evaluation indicators to explore the effects of UHPJ on the structure of casein. The results showed that with an increase of pressure, the free sulfhydryl group content changed irregularly, while the disulfide bond content increased from 1.085 to 3.0944 μmol/g. The content of α-helix and random coil in the casein decreased, while the β-sheet content increased at 100, 150, 200 MPa pressure. However, treatment with higher pressures of 250 and 300 MPa had the opposite effect. The average particle size of the casein micelles first decreased to 167.47 nm and then increased up to 174.63 nm; the absolute value of Zeta potential decreased from 28.33 to 23.77 mV. Scanning electron microscopy analysis revealed that the casein micelles had fractured into flat, loose, porous structures under pressure instead of into large clusters. After being ultra-high-pressure jet-processed, the sensory properties of skimmed milk and its fermented curd were analyzed concurrently. The results demonstrated that UHPJ could alter the viscosity and color of skimmed milk, shortening curdling time from 4.5 h to 2.67 h, and that the texture of the curd fermented with this skimmed milk could be improved to varying degrees by changing the structure of casein. Thus, UHPJ has a promising application in the manufacture of fermented milk due to its ability to enhance the curdling efficiency of skimmed milk and improve the texture of fermented milk.

## 1. Introduction

Ultra-high-pressure jet (UHPJ) processing is a new non-thermal processing technology. It divides liquids into two or more streams for Y-type, Z-type, or vertical impact [1]. The impact process combines multiple mechanical forces, such as high-velocity impact, high-frequency vibration, instantaneous pressure drop, intense shear, cavitation, and ultra-high pressure, and can generate a dynamic pressure of up to 200 MPa in a short period. Consequently, UHPJ processing can facilitate the homogenization of liquid materials, physical modification, and auxiliary sterilization. 

Studies have been conducted to investigate the effects of UHPJ processing on the structural and functional characteristics of proteins derived from food, such as ovalbumin [2], isolated soybean protein [3], peanut protein isolate [4], and myofibrillar protein [5]. The results indicate that UHPJ processing conducted at a relatively low pressure can partially unfold and denature part of a protein structure, leading to a decrease in the particle size and a changing in its secondary structure. As a result, proteins transition from an ordered state to a disordered one, while their structure loosens and the surface hydrophobicity increases. With an increase of pressure or treatment time, a protein will refold and aggregate, resulting in an increase in the particle size and a decrease in the surface hydrophobicity. In addition, UHPJ processing can enhance protein solubility [6]. At present, the research is focused on the alteration of high-purity food-derived proteins using UHPJ processing, though a few studies have explored the changes of unpurified proteins in raw materials using UHPJ processing.

Bovine milk, which is rich in protein, fat, vitamins, and minerals, is an important source of nutrition for humans. Accounting for approximately 80% of its total protein content, casein is the most abundant protein in milk and is divided into four main types: αs_1_-casein, αs_2_-casein, β-casein, and κ-casein [7]. Given that UHPJ processing can be applied to dairy manufacture [8,9], it is necessary to investigate the changes of casein in milk after UHPJ processing, and the potential effects of such processing on subsequent dairy processing. In this study, skimmed milk was treated using UHPJ processing at 100–300 MPa. Subsequently, the effects of the pressure on the structure of casein in skimmed milk and the changes of the sensory properties of skimmed milk and skimmed milk curd were studied. This provided a theoretical reference for the application of UHPJ technology in dairy processing.

## 2. Results and Discussion

### 2.1. Effect of UHPJ Processing on Temperature of Skimmed Milk

**Figure 1:**
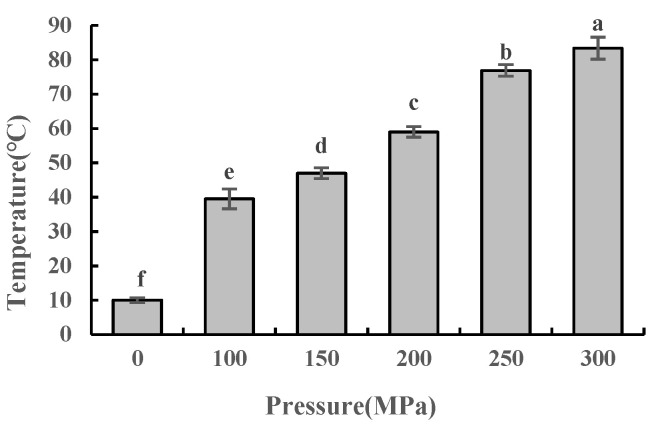
Sampling temperature of skimmed milk under different pressure conditions. Different superscript letters within a column indicate significant differences between means (*p* < 0.05).

As shown in Figure 1, the sampling temperature of skimmed milk rose rapidly with the increase in pressure. The injection temperature of the skimmed milk in this study was 10 °C. When the treatment pressure increased from 100 MPa to 300 MPa, the sampling temperature gradually increased from 39.5 ± 2.9 °C to 83.4 ± 3.2 °C. The reasons for this increase in temperature include ultra-high pressure, intense friction caused by shear, and the vaporization of milk when forced through a narrow reaction chamber microchannel [10]. This demonstrates that the UHPJ processing can increase the temperature of skimmed milk, which may be the consequence of high-velocity impact, high-frequency vibration, intense shear, or cavitation in addition to adiabatic heating generated by the machine [11].

### 2.2. Effect of UHPJ Processing on the Composition of Skimmed Milk

The composition of skimmed milk was determined by the Milko Scan FT-120. The data in Table 1 shows that the UHPJ processing had no significant effect on the composition of the skimmed milk, thus indicating that the UHPJ processing will not cause the decomposition of nutrients. 

### 2.3. Effect of UHPJ Processing on Content of Free Sulfhydryl and Disulfide Bonds in Casein

**Figure 2:**
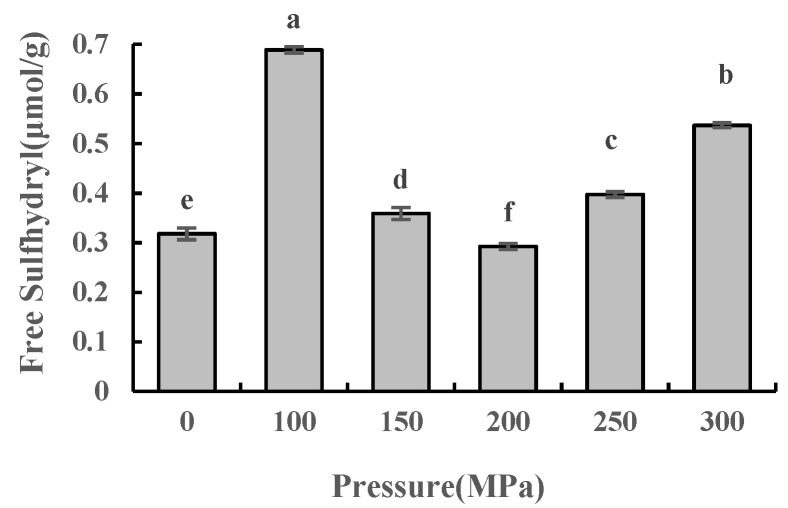
Free sulfhydryl content of casein under different pressure conditions. Different superscript letters within a column indicate significant differences between means (*p* < 0.05).

**Figure 3:**
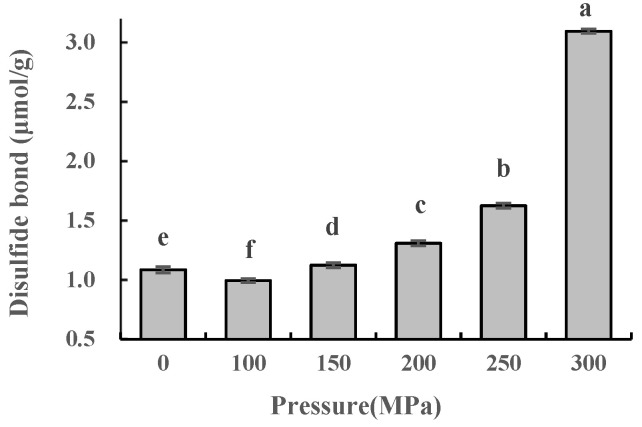
Disulfide bond content of casein under different pressure conditions. Different superscript letters within a column indicate significant differences between means (*p* < 0.05).

Free sulfhydryl and disulfide bonds are important chemical bonds that stabilize the conformation of protein molecules and are critical in determining the functional properties of proteins [12]. Treatments such as high pressure and heating can cause changes in the content of the free sulfhydryl and disulfide bonds in proteins. As shown in Figure 2 and Figure 3), the content of the free sulfhydryl in casein increased initially but then decreased after the high-pressure jet treatment. This content was significantly higher than that of the control group except for the sample at 200 MPa (*p* < 0.05). The content of the disulfide bond decreased at first but then increased and was significantly higher than that of the control group (*p* < 0.05) except for the 100 MPa sample; this is consistent with results from Wang et al. [13]. Under 100 MPa pressure, the content of the free sulfhydryl group in casein was 0.6887 μmol/g, which was significantly higher than that of the control group (*p* < 0.05). This may be attributed the strong shear and impact of the high-pressure jet, which destroyed the molecular structure of casein, exposing the sulfhydryl groups buried in αs_2_-casein [14] and κ-casein [15] molecules. The production rate of the free sulfhydryl group was higher than that of the oxidation, thus resulting in an increase in the content of the free sulfhydryl group (*p* < 0.05) and a decrease in the content of the disulfide bond (*p* < 0.05). The oxidation of the sulfhydryl groups was dominant at 150–300 MPa. The surface of the newly formed casein particles was thermodynamically unstable and some of the sulfhydryl groups were folded and embedded into the casein molecules. Therefore, compared with a treatment pressure of 100 MPa, the free sulfhydryl group content of casein decreased significantly (*p* < 0.05) while the disulfide bond content increased significantly (*p* < 0.05).

The content of the disulfide bond in casein increased more under 200–300 MPa pressure, which may be attributed to the denaturation of β-lactoglobulin owing to the higher pressure and temperature, and then formed an intermolecular disulfide bond with κ-casein [16]. The content of the disulfide bond reached its maximum value at 300 MPa, and simultaneously, the sensory evaluation experiment results showed that the cooking flavor of skimmed milk was also at its strongest (data not shown), indicating that an excessively high pressure was not conducive to preserving the milk’s unique flavor.

### 2.4. Effect of UHPJ Processing on Secondary Structure of Casein

The direction of the hydrogen bond in an α-helix is consistent with that of the helix axis, consequently it has strong rigidity, while the direction of hydrogen bond in a β-sheet is perpendicular to the direction of folding, consequently it has better flexibility. The stability of these secondary structures is different when subjected to the UHPJ processing and thermal treatment. The effect of the UHPJ processing on the content of the casein secondary structure is shown in Table 2. After pressure treatments of 100, 150, and 200 MPa, the content of the α-helix decreased significantly (*p* < 0.05), which may be attributed to the fact that the hydrogen bonds which maintain the structural stability of casein are partially broken under the action of a high-pressure jet, leading to the extension of the casein. Such a significant increase in the content of the α-helix (*p* < 0.05) under 250 and 300 MPa pressure may be due to the synergistic effect of high temperature and pressure, which enhances the interaction between casein molecules, changing the orientation of the hydrogen bonds in casein to generate a greater amount of them [17]. There was no significant difference in the content of the β-turn (*p* > 0.05) under different pressure conditions. The content of the random coil first decreased and then increased, while the content of the β-sheet changed in the opposite direction. In summary, the content of the α-helix and the random coil of casein decreased, yet the content of the β-sheet increased, when treated with the UHPJ at lower pressures (100, 150, 200 MPa), while the effect of UHPJ treatment with a higher pressure (250, 300 MPa) had the opposite effect.

### 2.5. Effect of UHPJ Processing on Average Particle Size of Casein Micelle

**Figure 4:**
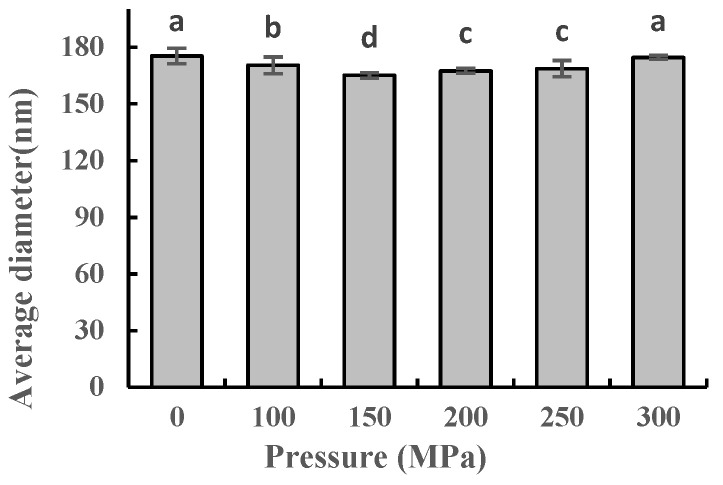
Average particle size of casein under different pressure conditions. Different superscript letters within a column indicate significant differences between means (*p* < 0.05).

The average particle size of the casein micelles under different pressures is shown in Figure 4. The average particle size decreased first but then increased with an increase in the treatment pressure, which is consistent with the research results from Mohan et al. [18]. At the relatively low pressures of 100 MPa and 150 MPa, the decrease in average casein particle size was due to external forces such as impact force, strong shear, and the void effect acting on the casein in the reaction chamber of the UHPJ homogenizer. The casein particles were crushed and the micelle structure was destroyed such that the average particle size was reduced. The average particle size of the casein micelles increased under pressures of 200–300 MPa, with no significant difference in average particle size between the sample at 300 MPa and the control. The reasons for this phenomenon are as follows: (1) The dissolution of κ-casein on the surface of the casein micelles under high pressure reduced the spatial repulsion between the micelles and the negative charge on the surface, thus promoting aggregation between the micelles [19]. (2) In the process of using the UHPJ, an increase of pressure was accompanied by an increase of temperature. β-lactoglobulin will denature when the pressure reaches 200 MPa or the temperature is 75–80 °C. As shown in Figure 1, the sample temperature of skimmed milk under 250 MPa pressure treatment reached 76.9 °C. The modified β-lactoglobulin interacted with the surface of the casein micelles, binding to increase the average particle size of the micelles [20].

### 2.6. Effect of UHPJ Processing on Zeta Potential of Casein Micelles

**Figure 5:**
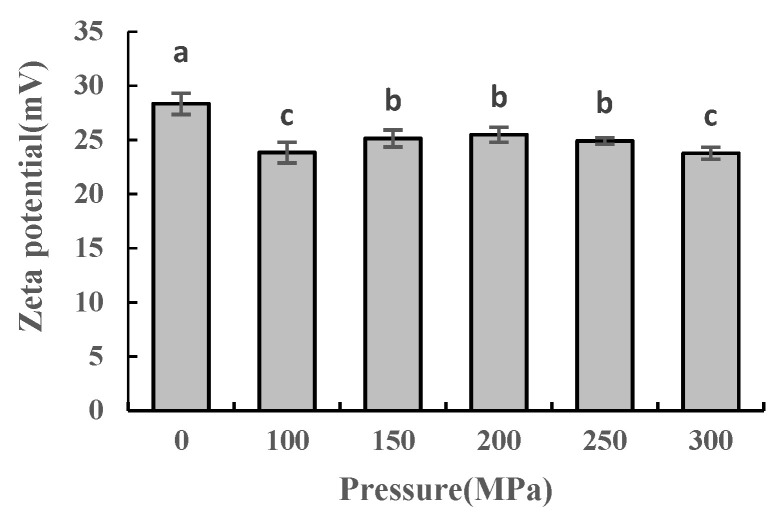
Zeta potential of casein under different pressure conditions. Different superscript letters within a column indicate significant differences between means (*p* < 0.05).

Zeta potential is the potential of the shear surface in a charged double layer of charged particles in solution [21], and it is one of the indicators used to evaluate the stability of colloids. The greater the absolute value of the Zeta potential, the greater the electrostatic repulsion force on the molecular surface and the more stable the casein colloid in the milk system. The smaller the absolute value of the Zeta potential, the more unstable the casein micelle. The absolute value of the Zeta potential of the samples in this study decreased after the pressure treatment compared with the control group, indicating that the stability of casein micelles also decreased (Figure 5). Janahar et al. [22] found that pressure-only treatments up to 400 MPa do not reduce the apparent particle size of whole milk’s viscosity, while ultra-shear technology (UST) or high-pressure homogenization facilitate both the particle size and the Zeta potential reduction. This suggests that shear is the dominant effect for the change of the particle size in ultra-high pressure processing.

Sandra [23] reported that the mechanism by which ultra-high-pressure homogenization (UHPH) modifies the structural properties of casein micelles in reconstituted skimmed milk powder is likely to be due to hydrophobic interaction or a shearing effect, while the application of heat treatment only has a minimal effect on the average casein micelle size. UHPH does not disrupt the casein micelles completely but rather dissociates parts of their surfaces. As a result, the protein complexes formed after UHPH are different from those formed after heating.

### 2.7. Effect of UHPJ Processing on the Microstructure of Casein

**Figure 6:**
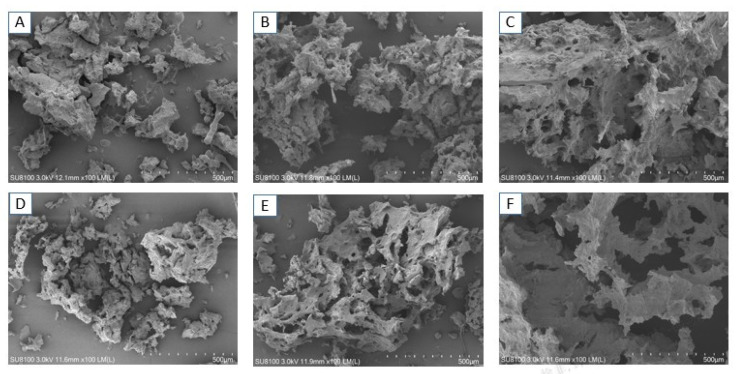
Scanning electron microscope diagram of casein under different pressure conditions. (**A**): 0 MPa (control); (**B**): 100 MPa; (**C**): 150 MPa; (**D**): 200 MPa; (**E**): 250 MPa; (**F**): 300 MPa; The magnification of scanning electron microscope was 100 times.

The microstructure of casein treated by UHPJ processing was observed by scanning electron microscope and the results are shown in Figure 6. Casein without the UHPJ processing showed an irregular and large cluster structure (Figure 6A). After being treated at 100 MPa (Figure 6B) and 150 MPa (Figure 6C) respectively, large clusters of casein were dispersed into small fluffy and porous clumps, which is consistent with the results of the particle size reduction. This shows that UHPJ processing can destroy protein structures and produce fragments of differing size and shape [24]. When the pressure exceeded 200 MPa, the casein micelle showed completely different structural characteristics, such as the formation of small casein clumps into aggregates. Casein aggregates formed at 250 MPa (Figure 6D) were larger than those formed at 200 MPa (Figure 6E), which is consistent with the results of the particle size increase. When the pressure reached 300 MPa, dense casein aggregates formed and the micelle structure flattened (Figure 6F). The above results suggest that protein depolymerization can be promoted by UHPJ processing at lower pressures, whereas protein re-aggregation can be initiated at higher pressures. Correspondingly, the casein particle size decreased at first and then increased, and the minimum particle size was observed at 150 MPa.

In addition, it has been demonstrated that colloidal calcium phosphate plays a critical role in maintaining the structural stability of the casein micelles [25]. High pressure can lead to the dissolution of colloidal calcium phosphate and a decrease in the particle size of the casein micelles.

### 2.8. Effect of UHPJ Processing on Apparent Viscosity of Skimmed Milk

**Figure 7:**
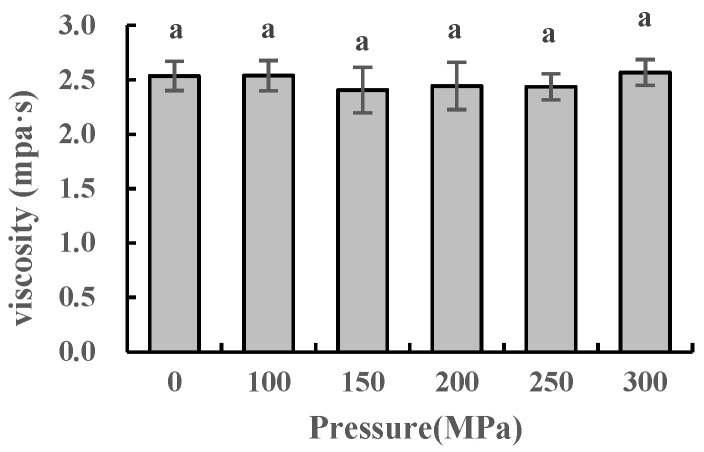
Apparent viscosity of skimmed milk under different pressure conditions. Different superscript letters within a column indicate significant differences between means (*p* < 0.05).

The change in apparent viscosity of the skimmed milk after the UHPJ processing is shown in Figure 7. The variation trend of apparent viscosity with pressure is consistent with that of the average particle size (Figure 4). Janahar et al. [22] reported that there is no significant difference between the viscosity of 400 MPa ultra-shear technology-treated whole milk and that of an untreated sample. Their results are similar to the viscosity of the sample treated with 300 MPa UHPJ in our experiment, although skimmed milk was used in our experiment. This phenomenon can be attributed to the disintegration of the original casein micelles under high pressure, which resulted in the formation of larger protein aggregates with a higher hydrodynamic volume [26].

### 2.9. Effect of UHPJ Processing on Skimmed Milk Color

The color of the object is typically expressed using a three-dimensional color model of *L*-*a*-*b* or L*-a*-b*, wherein L* represents brightness; a* represents the red-green system, with a* < 0 indicating the sample is greener than the standard; b* represents the yellow-blue system, with b* < 0 indicating the sample is bluer than the standard. At present, the color of milk and dairy products is mainly analyzed according to the relationship between the changes of L* and the changes of the milk protein structure, especially the particle size of the casein micelles. The color change of skimmed milk after the UHPJ processing is shown in Table 2. The smaller the value of L*, the lower the brightness of the skimmed milk, where L* is related to the state and the particle size of the casein. After being treated with UHPJ, the L* value of the skimmed milk decreased initially (100, 150 MPa) and subsequently increased (200–300 MPa). Usually, the variation of L* was consistent with that of the average particle size, which may be attributed to the destruction of the casein micelles and the formation of small fragments, thus increasing the transmittance of the milk [27,28]. In this study, there was no significant difference among all the samples (*p* > 0.05), which means the UHPJ processing had no significant effect on the brightness of skimmed milk.

The values of a* and b* for the samples both showed the trend of decreasing initially and then increasing, which is consistent with results from Pereda et al. [11]. This means that samples were much greener and bluer than the control under the pressures of 100–250 MPa, while samples were redder and yellower than the control at 300 MPa. However, there are few studies on the relationship between the change of a* and b* and the specific structural change of the components in skimmed milk.

The chromatic aberration value ΔE* presents overall color change, with a higher value of ΔE* indicating a greater overall color change between the sample and the control. According to the ΔE* data in Table 3, the color of all the samples changed to some extent, among which skimmed milk at 100 MPa showed the greatest difference in color compared to the control group.

### 2.10. Effect of UHPJ Processing on Coagulation Properties of Skimmed Milk

The particle size (Figure 4), secondary structure (Table 2), and microstructure (Figure 6) of the casein changed after the UHPJ processing; therefore, the processing properties of raw milk [28,29,30,31], such as the coagulation properties, emulsibility, and foaming properties, changed accordingly. In order to investigate the effects of the UHPJ processing on the follow-up processing of dairy products, skimmed yogurt was prepared from the skimmed milk that had been subjected to the UHPJ processing. The indexes related to the quality of the yogurt, such as the clotting time, apparent viscosity, and the water holding capacity, were evaluated and the results are shown in Table 4.

UHPJ processing above 150 MPa can shorten the curdling time of skimmed milk to varying degrees; for example, the clotting time of the samples treated with 250 and 300 MPa was 1.83 h shorter than that of the control group. In contrast to the change trend in the apparent viscosity of the unfermented skimmed milk treated by the UHPJ processing (Figure 7), the apparent viscosity and the viscosity of yoghurt prepared from this skimmed milk always increased with the increase of pressure. The water holding capacity (WHC) represents the ability of curd to retain water under the action of centrifugal, stirring, and other external forces. The WHC of skimmed yogurt increased continuously with an increase of pressure and reached its maximum when the pressure was 250 MPa, as reported by Serra et al. [28] and Ciron et al. [32]. The strength of the sample’s intermolecular binding was reflected by its cohesiveness, which increased as the pressure increased, suggesting that the UHPJ processing enhanced the interaction of the proteins in the skimmed yogurt. Firmness also kept rising with an increase in the pressure. When the pressure reached 250 MPa, however, it attained its extreme value. Even though firmness slightly declined when the pressure reached 300 MPa, it was still significantly higher than that of the control group.

The changes in texture properties observed above can be attributed to several factors. As the pressure increases, the casein micelles become smaller, which leads to an increase of effective surfaces with interaction forces and the formation of a strong gel network in skimmed yogurt [32]. Additionally, the temperature of raw milk at the outlet increased with the increase of pressure in the process of using the UHPJ (Figure 1). Under the combined effects of heating and pressure, the denaturation of the whey protein (β-lactoglobulin, immunoglobulins, and α-lactalbumin) intensified [28,29,30] and the solubility of the protein decreased, which also accelerated the coagulation of milk. Furthermore, the UHPJ process induced the formation of covalent disulfide bonds between casein and whey proteins, which contributed to the formation of a more compact gel and increased its water retention [33].

## 3. Materials and Methods

### 3.1. Materials and Reagents

For this study, fresh bovine milk was provided by the farm passing the GAP first-level certification (Tianjin, China). The chemical composition of raw milk is shown in Table 1. YoFlex^®^ Premium 1.0, the direct VAT inoculation composed of *Lactobacillus bulgaricus* and *Streptococcus thermophilus*, was provided by Chr. Hansen Holding A/S (Hoersholm, Denmark). Tris, glycine, ethylene diamine tetraacetic Acid (EDTA), dinitrobenzoic acid (DTNB), trichloroacetic acid (TCA), urea, and β-mercaptoethanol were all purchased from Beijing Solarbio Science & Technology Co., Ltd. All other chemicals and reagents used were of analytical grade.

### 3.2. Degreasing

**Figure 8:**
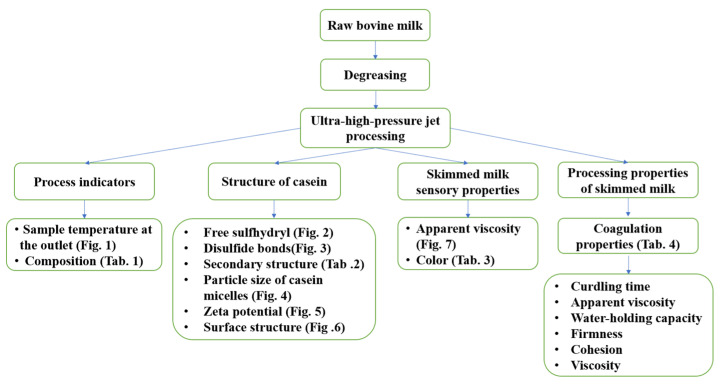
Flow chart of the experiment.

The preparation of the sample and the subsequent experimental process are shown in Figure 8. A pilot-type milk fat separator (GEA Group Co., Ltd., Bochum, Germany) was used for cold degreasing treatment of the fresh milk. The treatment flow was 450 L/h with a rotational speed of 9000–10,100 r/min.

### 3.3. UHPJ Processing

The milk was treated using ultra-high pressures of 100, 150, 200, 250 and 300 MPa (Dynamic ultra-high-pressure jet processing homogenizer, laboratory’s self-developed equipment, Tianjin, China) immediately after skimming. Each sample was processed only once. Samples were collected and cooled to 8 °C using a mixture of ice and water; then the samples were stored and refrigerated at 4 °C.

### 3.4. Analysis of Milk Composition

The composition of milk was determined by Milko Scan FT-120 (FOSS Analytical A/S, Denmark). Sample injection, testing and data analysis were controlled by the FT-120 software program, with an input temperature of 45 °C and injection volume of 30 mL.

### 3.5. Extraction of Bovine Casein

The skimmed milk treated by UHPJ processing was cooled to room temperature and its pH value was adjusted to 4.6 with a 1 mol/L HCl solution. Next, it was centrifuged at 4 °C and 7000 r/min for 15 min (high-speed freezing centrifuge 3K15, Sigma Laborzentrifugen GmbH, Osterode, Germany), after which the underlying precipitate was casein. This precipitate was washed several times with deionized water until it was neutral, then freeze-dried and preserved.

### 3.6. Determination of Sulfhydryl and Disulfide Bonds in Casein

The contents of the free sulfhydryl group and the disulfide bond of casein were determined according to Wang C.Y.’s [10] method.

For the determination of the free sulfhydryl content (SH_F_), 1 mL of casein solution with a concentration of 10 mg/mL was mixed with 5 mL of Tris-Gly-8M urea solution (0.086 mol/L Tris, 0.09 mol/L glycine, 0.004 mol/L EDTA and 8 mol/L urea) and 0.04 mL of DTNB solution (4 mg/mL), and then allowed to react at 25 °C for 30 min. The samples were monitored by measuring absorbance at 412 nm. The determination of the blank control was carried out using distilled water instead of casein.

For the determination of total sulfhydryl content (SH_T_), 1 mL of casein solution with a concentration of 10 mg/mL was mixed with 5 mL of Tris-Gly-10 M urea solution (0.086 mol/L Tris, 0.09 mol/L glycine, 0.004 mol/L EDTA and 10 mol/L urea) and 0.1 mL of β-mercaptoethanol, and then allowed to react at 25 °C for 1 h. Next, 10 mL of 12% TCA solution was added and this mixture was maintained at the same temperature for 1 h, then centrifugated at 3000 r/min for 10 min. After that, the precipitate was washed with 12% TCA solution and centrifuged for 15 min at 4000 r/min. This was repeated three times. The samples were monitored by measuring absorption at 412 nm, and distilled water was used for the control.

Sulfhydryl groups and disulfide bonds were calculated as follows:

(1) $$\mathrm{Sulfhydryl}\;\mathrm{group}\;\mathrm{content}\;\left(\mu \mathrm{mol}/g\;\mathrm{protein}\right)=\frac{73.53\times A_{412}}{C}$$

(2) $$\mathrm{Disulfide}\;\mathrm{bond}\;\mathrm{content}\;\left(\mu \mathrm{mol}/g\mathrm{protein}\right)=\frac{{\mathrm{SH}}_{T}-{\mathrm{SH}}_{F}}{2}$$

*A*_412_ was the absorbance at λ = 412 nm; *C* was the casein concentration of the sample in mg/mL.

### 3.7. Analysis of the Secondary Structure of Casein by Circular Dichroism

The secondary structure of casein was determined by circular dichroism spectrometer (MOS-500, Bio-Logic Science Instruments, Seyssinet-Pariset, France). The lyophilized casein powder was dissolved in a 0.02 mol/L phosphate buffer (pH 7.5) at a concentration of 0.1 mg/mL. Detection was carried out in the range of 190–260 nm with a step of 1 nm; one data point was collected every second. The same phosphate buffer was used as a blank. The secondary structure of each sample, including α-helix, β-fold, β-turning and random coil, was fitted and calculated by CDNN software to obtain the relative percentage content.

### 3.8. Determination of Particle Size and Zeta Potential of Casein

The particle size and Zeta potential of casein micelles in skimmed milk were determined by a laser nano-particle size potentiometer (Zetasizer Nano ZS, Malvern Panalytical, Malvern, UK). The skimmed milk was diluted 100 times with ultrapure water. The particle size of the diluent was measured at a diffraction angle of 173°. The test mode was a protein and the solution mode water. The Zeta potential of the same diluted skimmed milk was measured at room temperature. The test mode was automatic.

### 3.9. Surface Structure of Casein Mapped by Scanning Electron Microscope (SEM)

Following the method of Zhang A.Q. et al. [34], the microstructure of casein was observed with an SU8100 scanning electron microscope (Hitachi, Ltd., Tokyo, Japan). The freeze-dried casein powder was placed in a 1.5 mL centrifuge tube and fixed with glutaraldehyde for more than 4 h. Samples were washed 3 times at 10 min per time with a 0.1 mol/L phosphate buffer and were then fixed with 1% osmium tetroxide for 1 h.

The samples were gradient dehydrated with 50%, 70%, 80%, 90% and 100% ethanol for 10 min each time. The centrifugal tube was dried in a dryer for 12 h. Subsequently, the sample was taken out and adhered to a copper plate for conductive treatment. The sample was then observed by scanning electron microscope and the acceleration voltage was found to be 3 kV.

### 3.10. Measurement of Color of Skimmed Milk

Following the method employed by Chi X.L. et al. [35], a colorimeter (TS7700, Shenzhen ThreeNH Technology Co., Ltd., Shenzhen, China) was used to assess the color of skimmed milk after UHPJ processing. The Commission Internationale de l’Eclairage (CIE) L*, a* and b* of samples were measured with an illuminant of D65 at a standard 10°. ΔL*, Δa* and Δb*, which represent the differences between L*, a* and b* of each sample and the control, respectively, were calculated and subsequently the color difference value of each sample was calculated according to the following formula:

(3) $$\Delta E*\;=\sqrt{{\left(\Delta L^{*}\right)}^{2}+{\left(\Delta a^{*}\right)}^{2}+{\left(\Delta b^{*}\right)}^{2}}$$

### 3.11. Fermentation of Skimmed Milk

Skimmed milk treated by an UHPJ was inoculated with starter (YoFlex^®^ Premium 1.0, 0.02 U per 50 g of skimmed milk), shaken well, and fermented in a water bath at 42 °C until its pH reached 4.6. The curdling time of the sample was recorded. After the curd structure was destroyed by hand stirring, the curd was refrigerated at 4 °C for more than 4 h.

### 3.12. Properties of Skimmed Milk and Curd Texture

The apparent viscosity of skimmed milk and that of curd was measured with a rheometer (MCR302, Anton Paar, Graz, Austria). Then a texture analyzer was used to determine the curd’s hardness, cohesiveness, and viscosity (TA-XT Plus, Stable Systems Co., Ltd., U.K.). Texture measurement conditions were as follows: probe: A/BE35; Test distance: 25.0 mm; Probe speed: 2.0 mm/s; Inductive force: Auto-10.0 g. Each of the samples was measured 3 times.

### 3.13. Water-Holding Capacity of Curd

An amount of 25 g of the curd sample was weighed and recorded as *W*_1_. After being centrifuged at 4500 r/min (3K15, Sigma Laborzentrifugen GmbH, Osterode, Germany) for 15 min, the curd rested for 10 min. The supernatant was removed, after which the weight of the lower precipitate was measured and recorded as *W*_2_.

The water holding capacity of curd was calculated according to the following formula:

water holding capacity (%) = (*W*_2_/*W*_1_)
× 100% (4)

### 3.14. Data Processing and Analysis

The experiment was conducted three times to obtain all data, the outcomes of which were expressed as means ± SD. The significance threshold for the statistical analysis was set to 0.05 using SPSS 19.0.

## 4. Conclusions

The results of this study show that UHPJ processing not only alters the secondary and tertiary structures of casein, but also reduces the average particle size and Zeta potential of casein in skimmed milk, while also inducing changes in the microstructure of casein. The combination of these changes affects sensory properties, such as the color and viscosity of skimmed milk. At the same time, the curdling time of skimmed milk was effectively shortened, and the coagulation properties, such as the water holding capacity and the viscosity were significantly increased. As a result, UHPJ processing, which combines homogenization and sterilization functions, has prominent advantages for the raw milk pretreatment of yogurt, and these can be applied in dairy manufacture to improve the fermentation efficiency and the texture of fat-free yogurt.

However, the change in the coagulation property is the result of the combination of changes in both the casein and the whey proteins. Further research, which is currently being carried out by our team, will focus on the evaluation of the effects of the UHPJ processing on whey protein.

## Figures and Tables

**Table 1:** The composition of samples.

	Protein (%)	Fat (%)	Lactose (%)	SNF (%)	TS (%)
Raw milk	3.37 ± 0.01	4.04 ± 0.02	4.73 ± 0.01	9.18 ± 0.02	12.98 ± 0.02
Skimmed milk	3.01 ± 0.00 ^a^	0.11 ± 0.03 ^a^	4.91 ± 0.00 ^a^	9.76 ± 0.01 ^a^	9.82 ± 0.02 ^a^
100 MPa	3.01 ± 0.02 ^a^	0.11 ± 0.02 ^a^	4.91 ± 0.03 ^a^	9.76 ± 0.02 ^a^	9.82 ± 0.02 ^a^
150 MPa	3.01 ± 0.03 ^a^	0.11 ± 0.01 ^a^	4.91 ± 0.02 ^a^	9.76 ± 0.01 ^a^	9.82 ± 0.02 ^a^
200 MPa	3.01 ± 0.02 ^a^	0.11 ± 0.02 ^a^	4.91 ± 0.01 ^a^	9.76 ± 0.02 ^a^	9.82 ± 0.02 ^a^
250 MPa	3.01 ± 0.02 ^a^	0.11 ± 0.02 ^a^	4.91 ± 0.02 ^a^	9.76 ± 0.02 ^a^	9.82 ± 0.02 ^a^
300 MPa	3.01 ± 0.02 ^a^	0.11 ± 0.02 ^a^	4.91 ± 0.02 ^a^	9.76 ± 0.03 ^a^	9.82 ± 0.02 ^a^

Results are presented as mean ± SD for triplicate samples. SNF: non-fat milk solid. TS: total solids. Different superscript letters within a column indicate significant differences between means (*p* < 0.05).

**Table 2:** Content of secondary structural components of casein under different pressure conditions.

Pressure/MPa	Content of Secondary Structure Components of Casein (%)			
	α-Helic	β-Sheet	β-Turn	Random Coils
0	12.22 ± 0.23 ^c^	26.92 ± 0.35 ^b^	25.07 ± 0.04 ^a^	36.14 ± 0.22 ^c^
100	11.14 ± 0.18 ^f^	27.35 ± 0.11 ^b^	24.86 ± 0.08 ^a^	34.75 ± 0.17 ^e^
150	11.73 ± 0.32 ^d^	28.41 ± 0.25 ^a^	25.19 ± 0.12 ^a^	35.09 ± 0.13 ^de^
200	11.48 ± 0.09 ^e^	28.95 ± 0.05 ^a^	24.65 ± 0.22 ^a^	35.52 ± 0.19 ^d^
250	14.39 ± 0.15 ^a^	24.63 ± 0.18 ^c^	24.53 ± 0.14 ^a^	37.07 ± 0.16 ^b^
300	13.21 ± 0.21 ^b^	23.18 ± 0.16 ^d^	24.71 ± 0.13 ^a^	39.03 ± 0.21 ^a^

Results are presented as mean ± SD for triplicate samples; different superscript letters within a column indicate significant differences between means (*p* < 0.05).

**Table 3:** Color change of skimmed milk under different pressures.

Pressure/MPa	L*	a*	b*	ΔE*
0	70.60 ± 0.29 ^a^	−7.22 ± 0.07 ^a^	−5.12 ± 0.07 ^b^	/
100	70.61 ± 0.29 ^a^	−7.79 ± 0.27 ^b^	−6.57 ± 0.27 ^d^	1.57 ± 0.31 ^a^
150	70.80 ± 0.12 ^a^	−7.74 ± 0.05 ^b^	−5.74 ± 0.12 ^c^	0.90 ± 0.00 ^b^
200	70.50 ± 0.25 ^a^	−8.01 ± 0.10 ^b^	−5.55 ± 0.30 ^bc^	0.91 ± 0.27 ^b^
250	70.60 ± 0.33 ^a^	−7.87 ± 0.06 ^b^	−5.33 ± 0.06 ^bc^	0.68 ± 0.02 ^b^
300	70.59 ± 0.39 ^a^	−6.89 ± 0.13 ^a^	−4.16 ± 0.22 ^a^	1.03 ± 0.20 ^b^

Results are presented as mean ± SD for triplicate samples; different superscript letters within a column indicate significant differences between means (*p* < 0.05).

**Table 4:** Coagulation properties of skimmed milk after different pressure treatments.

Pressure/ MPa	Curding Time/h	Apparent Viscosity/mPa·s	Water Holding Capacity/%	Firmness/g	Cohesive/g	Viscosity/g.Sec
0	4.50 ± 0.08 ^a^	70.72 ± 0.12 ^f^	24.51 ± 1.1 ^d^	6.21 ± 0.56 ^d^	0.79 ± 0.53 ^d^	1.19 ± 0.78 ^e^
100	4.50 ± 0.22 ^a^	131.93 ± 0.16 ^d^	25.77 ± 0.95 ^c^	6.36 ± 0.37 ^c^	0.81 ± 0.42 ^bc^	1.21 ± 0.43 ^cd^
150	3.75 ± 0.15 ^b^	130.81 ± 0.09 ^de^	26.86 ± 1.5 ^b^	6.35 ± 1.2 ^c^	0.87 ± 0.86 ^b^	1.39 ± 0.39 ^c^
200	3.33 ± 0.05 ^bc^	148.16 ± 0.13 ^c^	26.80 ± 1.3 ^b^	6.33 ± 0.81 ^c^	0.85 ± 0.94 ^b^	1.43 ± 1.2 ^c^
250	2.67 ± 0.8 ^d^	286.38 ± 0.21 ^b^	35.31 ± 0.68 ^a^	6.86 ± 0.85 ^a^	1.07 ± 0.68 ^a^	1.95 ± 0.94 ^b^
300	2.67 ± 0.13 ^d^	419.95 ± 0.25 ^a^	35.85 ± 0.84 ^a^	6.61 ± 0.62 ^b^	1.10 ± 0.83 ^a^	2.39 ± 1.7 ^a^

Results are presented as mean ± SD for triplicate samples; different superscript letters within a column indicate significant differences between means (*p* < 0.05).

## Data Availability

Data are contained within the article.
